# Tuberculosis preventive treatment and care cascade among household contacts of patients starting tuberculosis treatment in Southern Africa: a multicountry cohort analysis

**DOI:** 10.1136/bmjgh-2026-025497

**Published:** 2026-10-30

**Authors:** Leticia Wüthrich, Guy Muula, Denise Evans, Jacqueline Huwa, Cordelia Kunzekwenyika, Idiovino Rafael, Fiona Mureithi, Nelly Jinga, Amina Fernando, Agness Thawani, Marie Ballif, Lukas Fenner, Nicolas Banholzer, on behalf of IeDEA Southern Africa (IeDEA-SA)

**Affiliations:** 1 Institute of Social and Preventive Medicine (ISPM), University of Bern, Bern, Switzerland; 2 Centre for Infectious Disease Research in Zambia (CIDRZ), Lusaka, Zambia; 3 Health Economics and Epidemiology Research Office, Faculty of Health Sciences, University of the Witwatersrand, Johannesburg, South Africa; 4 Lighthouse, Lilongwe, Malawi; 5 SolidarMed, Masvingo, Zimbabwe; 6 SolidarMed, Chiure, Mozambique; 7 Department of Infectious Diseases, Inselspital, Bern University Hospital, University of Bern, Bern, Switzerland; 8 Department of Public Health, University of Copenhagen, Copenhagen, Denmark

**Keywords:** Tuberculosis

## Abstract

**Background:**

Tuberculosis preventive treatment (TPT) is recommended for household contacts of tuberculosis (TB) patients, yet implementation gaps persist, particularly in high-burden settings. We evaluated the TB preventive care cascade and determinants of TPT initiation among household contacts of patients with TB across five Southern African countries.

**Methods:**

In a prospective cohort study within the International Epidemiology Database to Evaluate AIDS Southern Africa (IeDEA-SA), we enrolled all individuals aged ≥ 15 years with pulmonary TB starting treatment between October 2022 and March 2023 at five TB/HIV care clinics in Malawi, Mozambique, South Africa, Zambia and Zimbabwe. We analysed the preventive care cascade of their household contacts along sequential outcomes: healthcare visit, TB symptom reporting, microbiological testing, diagnosis and TPT initiation and completion. Determinants of TPT initiation were assessed using multivariable logistic regression.

**Results:**

Among 1438 index patients with TB, 1369 (95%) reported at least one household contact, yielding 5805 contacts: 4529/5805 (78%) visited a healthcare facility and 877/4529 (19%) reported current TB symptoms, 297/4529 (7%) tested for TB, which was confirmed, latent or suspected in 74/4529 (2%). Among 4455 contacts visiting a healthcare facility with no confirmed, suspected or latent TB, only 223 (5%) initiated TPT, more frequently among people with HIV (OR 3.28, 95% CI 1.78 to 6.02) and children under 5 years (OR 3.27, 95% CI 1.42 to 7.54). At study end, 137 (61%) completed TPT, 84 (38%) were still on treatment, only 1 contact interrupted treatment and 1 had unknown status.

**Conclusion:**

Despite high healthcare engagement among index patients, TPT initiation among household contacts in Southern Africa was low although relatively higher among priority groups such as people with HIV and young children. Strengthening the link between household contact screening and TPT uptake is essential to close the preventive care cascade gap in the region.

**WHAT IS ALREADY KNOWN ON THIS TOPIC**

- Tuberculosis preventive treatment (TPT) is recommended and effective for household contacts of people with tuberculosis (TB), yet global coverage remains far below the 90% United Nations target, and initiation rates in Southern Africa frequently fail to reach even 40% of eligible people, with particularly low coverage among children under 5 years.

**WHAT THIS STUDY ADDS**

- In a prospective cohort of 5805 household contacts across five Southern African countries, 78% visited a healthcare facility but only 5% of eligible contacts initiated TPT, confirming a critical implementation gap despite high healthcare engagement.
- TPT initiation was about three times more likely among priority groups, including people with HIV and children under 5 years, and was also higher among symptomatic contacts.

**HOW THIS STUDY MIGHT AFFECT RESEARCH, PRACTICE OR POLICY**

- Closing the preventive cascade gap will require strengthening the link between household contact screening and TPT initiation through integrated TB/HIV services, household-centred and community-based contact tracing, improved patient counselling and health education and the use of short-course regimens.

## Introduction

 Tuberculosis (TB) remains one of the world’s leading causes of death among infectious diseases, with the Southern African region bearing a disproportionate share of this burden.^1^ Despite significant progress, the region faces high rates of TB incidence and mortality, often fuelled by the dual epidemic of HIV,^1^ TB/HIV service disruptions during the COVID-19 pandemic^2^ and recent global US funding cuts for TB.^3^ To end the TB epidemic, the ‘end TB Strategy’ of the WHO emphasises a comprehensive approach that includes not only the diagnosis and treatment of active disease but also the systematic prevention of new cases.^4^ A critical component of this strategy is the TB preventive care cascade, a framework used to identify and measure gaps in care from the identification of high-risk individuals and screening for active disease to the initiation and completion of TB preventive treatment (TPT).^4^ Contact investigations improve detection of people with TB and TB treatment outcomes,^5^ also through subsequent TPT, which has been shown to effectively prevent TB, especially in high-burden settings.^6 7^ Further modelling work suggests that TPT is also cost-effective.^8 9^

TPT is a course of treatment administered to individuals at high risk of developing active TB disease following infection or exposure to *Mycobacterium tuberculosis (Mtb*). Current WHO guidelines prioritise several groups for TPT^10^: people with HIV, household contacts of bacteriologically confirmed pulmonary TB cases (regardless of age but prioritising children <5 years) and individuals with other clinical risk factors such as silicosis or those initiating immunosuppressive therapy. Global targets set by the United Nations High-Level Meeting on TB aimed to provide TPT to 30 million people between 2018 and 2022, including 20 million household contacts.^11^ Most clinics reported offering TPT to at-risk populations^12^ and a meta-analysis estimated that 40%–60% of people with HIV initiated TPT.^13^ However, global coverage remains below the 90% target set by the United Nations’ High-Level Meeting in 2023,^14^ and initiation rates in Southern Africa frequently fail to reach even 40% of the eligible population and coverage among children under 5 years is low.^15–18^

Household contact tracing represents a promising yet underused approach for identifying and initiating TPT.^19^ Extant literature from lower-income and middle-income countries identifies a range of structural and sociodemographic barriers that hinder TPT initiation among household contacts. Studies in Southern Africa have shown that clinical barriers, such as the need to exclude active TB and the historical reliance on long 6-to-12-month isoniazid regimens, contribute to high attrition rates.^16 20 21^ Furthermore, sociodemographic factors play a significant role; for instance, lower TPT initiation was found for children and adolescents^16 22 23^ and people with lower education or income.^17 23^ Characteristics of the index patient, such as their HIV status, the severity of their disease and the nature of the relationship to the contact may also play a role, but there is a lack of multicountry data examining how these characteristics influence the TPT care cascade.

This study addresses these gaps by evaluating the TB preventive care cascade within the International Epidemiology Database to Evaluate AIDS Southern Africa (IeDEA-SA) network across five countries: South Africa, Malawi, Zambia, Zimbabwe and Mozambique. By collecting data from household contacts of index patients starting TB treatment, we aim to quantify the proportion of contacts who visited a healthcare facility, were diagnosed with active TB or initiated (and completed) TPT. We also examined how the characteristics of the contact and index patient were associated with the TB preventive care cascade. Our findings seek to provide actionable evidence to optimise contact tracing and preventive care delivery in low-resource settings with high TB burden.

## Methods

### Study setting

In a prospective cohort study, we enrolled all individuals ≥15 years with pulmonary TB starting TB treatment between September 2022 and April 2024 in one of the five TB/HIV care clinics in IeDEA-SA (not all sites started recruitment at the same time): Lighthouse and Martin Preuss Center, Lilongwe (Malawi); Chiure Health Center, Chiure (Mozambique); Themba Lethu Clinic, Helen Joseph Hospital, Johannesburg (South Africa); Centre for Infectious Disease Research in Zambia (CIDRZ), Lusaka (Zambia); SolidarMed-supported health facilities in Bikita and Zaka districts, Masvingo province (Zimbabwe). TB was diagnosed either through bacteriological confirmation (eg, Xpert MTB/RIF Ultra or urine lipoarabinomannan tests) or through clinical diagnosis based on symptoms and chest X-ray findings. We included data on TB treatment outcomes up until May 2025.

### Contact tracing

Household contact information was collected via index patients, that is, people diagnosed with TB and enrolled in the cohort. The index patients were given a short leaflet listing the information that would be needed about their household contacts during an in-depth follow-up interview of the index patients with a trained healthcare professional, or the index patients were given one form per household contact that they could fill out at home together with their household contacts and bring back at their next visit. If the index patients did not return for their next visit or follow-up interview, a contact tracing team was sent out. Information was captured via two standardised forms and entered electronically into the REDCap database by trained healthcare professionals. The first form recorded sociodemographic and clinical information for each household contact. This form was completed by the index patient at the start of TB treatment (baseline), end of treatment as well as 6 and 12 months post-treatment. The second form documented the contact tracing activities by the clinic of the index patient. This form was completed by the trained healthcare professionals and only at the baseline visit.

### Definitions

We refer to people with TB enrolled in the study as index patients and their household members as contacts. A household contact was defined as any person who shared the same enclosed living space as the index patient for one or more nights, or for frequent or extended daytime periods, within the 3 months before the start of the index patient’s current TB treatment.^10^ A contact was considered to have had a healthcare visit if the index patient reported that the contact had recently visited a healthcare facility or if there was any other evidence that the contact had visited one (eg, microbiological testing, TB diagnosis or TPT initiation). For this study, we considered household contacts eligible for TPT if they had any healthcare visit at which they were *not* diagnosed with TB; contacts with confirmed, latent or suspected TB were not considered eligible for TPT. Throughout the manuscript, ‘tested for TB’ refers to microbiological TB testing, while a diagnosis of active TB could also be made without microbiological confirmation. For TPT, we defined two priority groups: people with HIV and children <5 years old.

We included multiple characteristics of the household contact and index patient possibly associated with steps in the TB preventive care cascade. We dichotomised age as <5 or ≥5 years, as children under 5 years old are a WHO priority group for TPT. Education was categorised as no formal education, primary education and secondary or higher education. Dwelling location was dichotomised as urban versus rural. A history of TB was defined as any previous episode of active TB disease. Bacteriologically confirmed TB was defined by a positive smear microscopy, culture or Xpert MTB/RIF assay, whereas clinically diagnosed TB was based on clinical or radiographic findings without laboratory confirmation. Multidrug-resistant TB (MDR-TB) was defined as resistance to at least rifampicin.

### Linking household contact information across timepoints

Index patients provided information on their household contacts at four visits: baseline, end of treatment, and 6 and 12 months post-treatment. While index patients had a unique study identifier, contacts did not. Therefore, we used an algorithmic procedure to link multiple entries from follow-up visits possibly belonging to the same household contact (see online supplemental text A for details). Entries were linked if the personal characteristics were highly similar, considering the date of birth, initials, sex and the relationship to the index patient. Entries were linked sequentially starting from the baseline visit and new contacts were created for entries that could not be linked to entries from previous visits. Some of these new contacts were potential mismatches because the new entry did not align with an increase in the number of household members reported by the index patient. Therefore, we performed a sensitivity analysis where we excluded these potential mismatches.

After linkage, we summarised contact information across time points as follows. Variables referring to the TB/HIV care cascade (healthcare visits, TB symptoms, microbiological testing, TB diagnosis, TB treatment, TPT initiation and completion) were collapsed by prioritising ‘yes’ responses over ‘no’ and ‘no’ over ‘unknown’, ensuring that the most clinically relevant status observed across all visits of the index patient was captured for each household contact.

### Statistical analysis

We analysed the TB preventive care cascade of household contacts along the following outcomes: if the contact (1) came for a healthcare visit, (2) reported current TB symptoms, (3) tested for TB, (4) was diagnosed with TB and (5) initiated TPT. We reported TPT completion only descriptively and we did not model TB treatment initiation as an outcome because all contacts diagnosed with TB except one started treatment. All other outcomes were modelled using multivariable logistic regression models with country fixed effects and standard errors clustered at the household level. Model predictors included the sociodemographic and clinical characteristics of the household contact, index patient and their relationship.

Records with missing outcome values were removed from each analysis. Missing predictor values were imputed using multiple imputation by chained equations, including all outcomes and predictors in the imputation model. We imputed contact-level variables separately from index-level variables, using index patient characteristics as predictors of contact-level variables, but not vice versa. We generated 20 imputed datasets and fitted the regression models to each dataset. The estimation results were pooled using Rubin’s rules and reported as odds ratios (ORs) with 95% confidence intervals (CIs) and p values. Continuous variables are reported as medians (interquartile ranges [IQRs]) and categorical variables as counts (proportions, %). All analyses were performed in R V.4.5.1.

### Patient and public involvement

We discussed the objectives, the content and the study design with the relevant working groups within the global IeDEA consortium, including the TB and Lung Health working groups. Patients or the public were not directly involved in the design, or conduct, or reporting, or dissemination plans of our research. We will disseminate the results of this study to the participating HIV clinics and our broader research networks.

## Results

### Contact tracing activities

Across five cohorts, 1438 index patients with TB were enrolled. Thereof, 509 (35%) were contacted through formal tracing, mostly in Mozambique (online supplemental table S1). After diagnosis, some form of contact tracing occurred for 831 (58%) of index patients, including their household members (820/831, 99%). The contacts of 1019 (71%) index patients could be reached to recommend visiting a healthcare facility.

### Study population

At least one household contact was reported by 1369/1438 index patients (95%), with a total of 5805 household contacts and a median of 4 contacts per index (IQR 2–6). Table 1 shows the characteristics of index patients and their household contacts. Among index patients, median age was 39 years (IQR 30–50), 34% were female, 21% with a history of TB, 40% with HIV and 4% with MDR-TB. Household contacts were 31% children, 16% siblings and 13% partners, while 37% were non-immediate relatives and 3% were non-relatives. Their median age was 18 years (IQR 9–34), 13% were young children <5 years, 52% female, 8% with HIV, 3% with a history of TB.

**Table 1:** Characteristics of (A) household contacts and (B) index patients

(A) Household contact characteristics						
Median (IQR) or count (%)	Overall N=5805	Malawi N=1118	Mozambique N=1113	South Africa N=479	Zambia N=1963	Zimbabwe N=1132
Age (years)	18 (9, 34)	18 (8, 29)	19 (9, 36)	27 (14, 41)	19 (9, 33)	15 (8, 34)
<5	503 (13%)	71 (17%)	91 (11%)	14 (5%)	215 (13%)	112 (15%)
≥5	3310 (87%)	343 (83%)	709 (89%)	242 (95%)	1382 (87%)	634 (85%)
Missing	1992	704	313	223	366	386
Sex						
Male	2469 (48%)	529 (51%)	397 (45%)	153 (48%)	892 (49%)	498 (46%)
Female	2673 (52%)	514 (49%)	486 (55%)	168 (52%)	928 (51%)	577 (54%)
Missing	663	75	230	158	143	57
History of TB						
No	3925 (97%)	981 (97%)	*	229 (92%)	1702 (96%)	1013 (98%)
Yes	139 (3%)	35 (3%)	*	21 (8%)	63 (4%)	20 (2%)
Missing	1741	102	1113	229	198	99
HIV status						
Negative	3557 (92%)	527 (88%)	351 (96%)	189 (81%)	1608 (94%)	882 (93%)
Positive	303 (8%)	73 (12%)	13 (4%)	43 (19%)	106 (6%)	68 (7%)
Missing	1945	518	749	247	249	182
Current TB symptoms						
No	4874 (84%)	995 (89%)	726 (65%)	443 (92%)	1606 (82%)	1104 (98%)
Yes	931 (16%)	123 (11%)	387 (35%)	36 (8%)	357 (18%)	28 (2%)
Healthcare visit						
No	1276 (22%)	81 (7%)	307 (28%)	218 (46%)	613 (31%)	57 (5%)
Yes	4529 (78%)	1037 (93%)	806 (72%)	261 (54%)	1350 (69%)	1075 (95%)
Tested for TB						
No	4232 (93%)	1026 (99%)	795 (99%)	249 (95%)	1105 (82%)	1057 (98%)
Yes	297 (7%)	11 (1%)	11 (1%)	12 (5%)	245 (18%)	18 (2%)
TB diagnosis						
Confirmed active	59 (1%)	12 (1%)	15 (2%)	2 (1%)	25 (2%)	5 (<1%)
Suspected active	6 (<1%)	1 (<1%)	0 (0%)	0 (0%)	2 (<1%)	3 (<1%)
Latent TB	9 (<1%)	0 (0%)	4 (<1%)	1 (<1%)	1 (<1%)	3 (<1%)
No TB	4455 (98%)	1024 (99%)	787 (98%)	258 (99%)	1322 (98%)	1064 (99%)
TPT initiation						
Yes	223 (5%)	29 (3%)	72 (9%)	21 (8%)	13 (1%)	88 (8%)
No	4232 (93%)	995 (96%)	715 (89%)	237 (91%)	1309 (97%)	976 (91%)
Not applicable	74 (2%)	13 (1%)	19 (2%)	3 (1%)	28 (2%)	11 (1%)
TB treatment						
Started	58 (1%)	12 (1%)	15 (2%)	2 (1%)	24 (2%)	5 (<1%)
Not applicable	4471 (99%)	1025 (99%)	791 (98%)	259 (99%)	1326 (98%)	1070 (>99%)
TPT status						
Completed	137 (3%)	27 (3%)	20 (3%)	18 (7%)	8 (1%)	64 (6%)
Ongoing	84 (2%)	2 (<1%)	51 (6%)	3 (1%)	5 (<1%)	23 (2%)
Interrupted	1 (<1%)	0 (0%)	0 (0%)	0 (0%)	0 (0%)	1 (<1%)
Not applicable	4306 (95%)	1008 (97%)	734 (91%)	240 (92%)	1337 (99%)	987 (92%)
Unknown	1	0	1	0	0	0
TB treatment status						
Completed	3 (<1%)	2 (<1%)	0 (0%)	0 (0%)	1 (<1%)	0 (0%)
Ongoing	51 (1%)	10 (1%)	14 (2%)	1 (<1%)	21 (2%)	5 (<1%)
Interrupted	3 (<1%)	0 (0%)	1 (<1%)	0 (0%)	2 (<1%)	0 (0%)
Not applicable	4471 (99%)	1025 (99%)	791 (98%)	259 (>99%)	1326 (98%)	1070 (>99%)
Unknown	1	0	0	1	0	0
Relationship to index						
Sibling	802 (16%)	187 (19%)	59 (7%)	65 (22%)	404 (22%)	87 (8%)
Child	1554 (31%)	334 (34%)	370 (42%)	73 (25%)	408 (23%)	369 (34%)
Partner	663 (13%)	132 (13%)	205 (23%)	27 (9%)	170 (10%)	129 (12%)
Other non-relative	160 (3%)	30 (3%)	0 (0%)	14 (5%)	97 (5%)	19 (2%)
Other relative	1875 (37%)	309 (31%)	250 (28%)	113 (39%)	731 (40%)	472 (44%)
Missing	751	126	229	187	153	56

(B) Index characteristics†						
Median (IQR) or count (%)	Overall N=1369	Malawi N=258	Mozambique N=300	South Africa N=196	Zambia N=360	Zimbabwe N=255
Age	39 (30, 50)	35 (27, 45)	47 (34, 58)	40 (34, 50)	34 (28, 42)	44 (34, 58)
Sex						
Male	906 (66%)	190 (74%)	168 (56%)	119 (61%)	274 (76%)	155 (61%)
Female	463 (34%)	68 (26%)	132 (44%)	77 (39%)	86 (24%)	100 (39%)
History of TB						
No	840 (79%)	199 (77%)	*	163 (83%)	264 (73%)	214 (84%)
Yes	229 (21%)	59 (23%)	*	33 (17%)	96 (27%)	41 (16%)
Missing	300	0	300	0	0	0
HIV status						
Negative	820 (60%)	159 (62%)	218 (74%)	75 (40%)	227 (63%)	141 (55%)
Positive	536 (40%)	98 (38%)	78 (26%)	113 (60%)	133 (37%)	114 (45%)
Missing	13	1	4	8	0	0
MDR-TB						
No	1316 (96%)	258 (>99%)	300 (>99%)	190 (97%)	320 (89%)	248 (97%)
Yes	53 (4%)	0 (0%)	0 (0%)	6 (3%)	40 (11%)	7 (3%)
Education						
No formal	262 (19%)	18 (7%)	215 (72%)	0 (0%)	19 (5%)	10 (4%)
Primary	390 (29%)	117 (45%)	72 (24%)	9 (5%)	108 (30%)	84 (33%)
Secondary or higher	710 (52%)	123 (48%)	12 (4%)	183 (95%)	232 (65%)	160 (63%)
Missing	7	0	1	4	1	1
TB diagnosis						
Bacteriological	865 (81%)	149 (58%)	*	181 (92%)	345 (96%)	190 (75%)
Clinical	204 (19%)	109 (42%)	*	15 (8%)	15 (4%)	65 (25%)
Missing	300	0	300	0	0	0
Household size	4 (2, 6)	4 (3, 6)	3 (2, 5)	2 (1, 3)	5 (3, 8)	4 (3, 6)
Household location						
Urban	786 (58%)	240 (93%)	0 (0%)	192 (98%)	339 (94%)	15 (6%)
Rural	580 (42%)	17 (7%)	300 (>99%)	3 (2%)	21 (6%)	239 (94%)
Missing	3	1	0	1	0	1

* Not systematically recorded in Mozambique and thus fully imputed for subsequent analyses.

† Characteristics of index patients with at least one recorded household contact; among 1438 index patients, 1369 (95%) had at least one recorded household contact.

MDR-TB, multidrug-resistant TB; TB, tuberculosis; TPT, tuberculosis preventive treatment.

### TB preventive care cascade

Figure 1A shows the TB preventive care cascade. Among the 5805 household contacts, 4529 (78%) visited a healthcare facility, of which 877 (19%) reported current TB symptoms, 297 (7%) tested for TB (microbiological testing), and TB was confirmed, latent or suspected in 74 (2%): 59 with confirmed active TB, 9 with latent TB and 6 with suspected TB. Those with diagnosed or suspected TB were, therefore, not considered eligible for TPT. Among the 4455 TPT-eligible contacts who had visited a healthcare facility, 223 (5%) initiated TPT, most of them without prior microbiological TB testing (194, 87%). By the end of the study period, 137 (61%) had completed TPT, 84 (38%) were still on treatment, and only 1 contact had interrupted treatment, while the status was unknown for 1 other contact.

**Figure 1:**
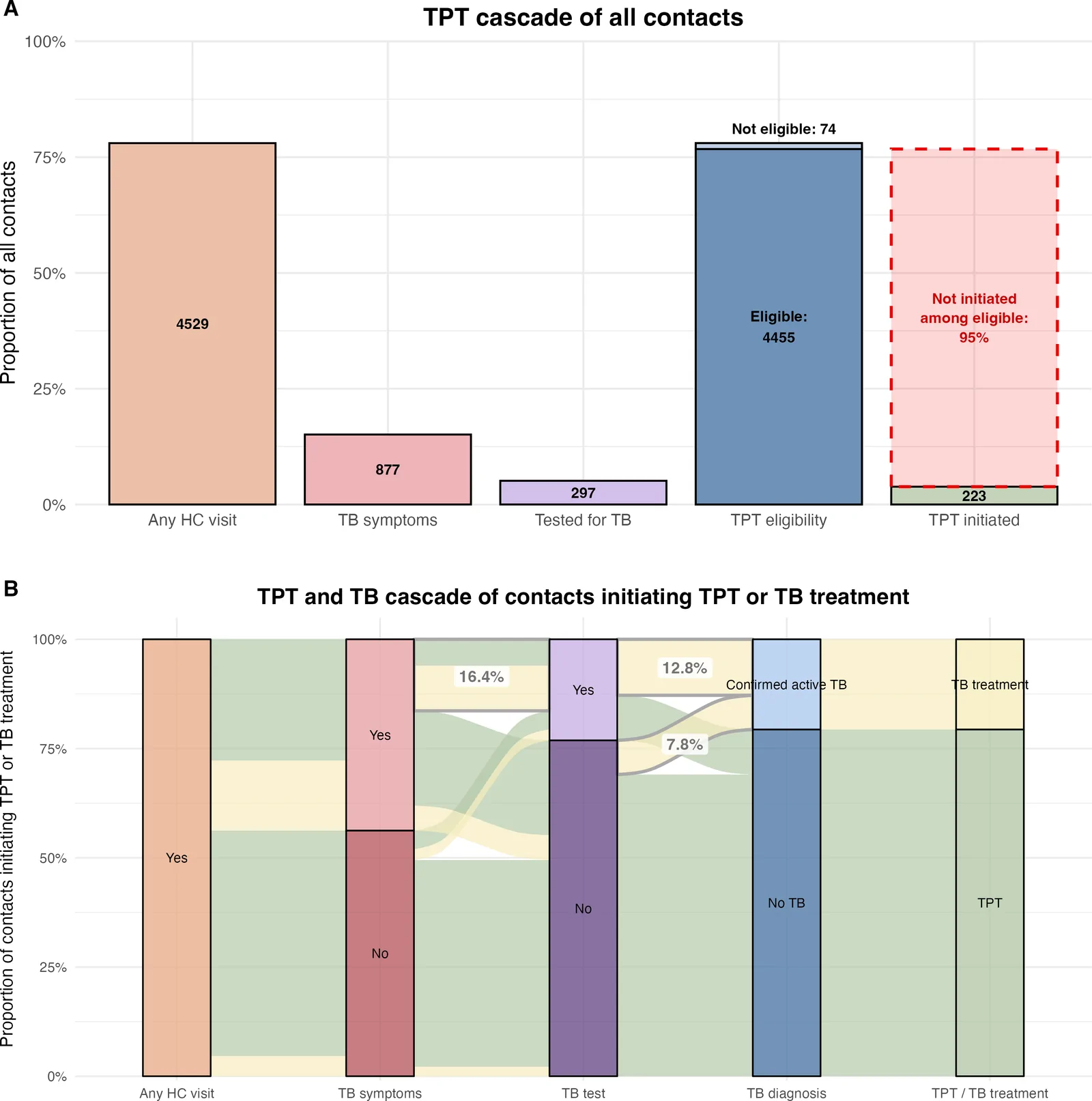
TB preventive care cascade among household contacts of index patients. (**A**) Proportion of all household contacts (N=5805) at each step of the TPT cascade, with the number of contacts shown in each bar. For TB symptoms, only contacts who also visited a healthcare facility are shown (877 of the 931 contacts reporting current symptoms; table 1). (**B**) Flows among only household contacts initiating TPT or TB treatment. Steps include visiting any healthcare facility (HC), reporting current TB symptoms, testing for TB (microbiological testing), receiving a TB diagnosis, and initiating TPT or TB treatment. TPT eligibility: all household contacts of people with TB visiting a healthcare facility were considered eligible for TPT, except contacts with confirmed, latent or suspected TB cases. TB tuberculosis; TPT tuberculosis preventive treatment.

Figure 1B highlights the TB preventive care cascade for the 223 contacts who initiated TPT and the 58/59 (98%) contacts who were diagnosed with active TB and started TB treatment. Among these 281 contacts, 123 (44%) had reported current TB symptoms and 46 (16%) had both reported current symptoms and tested for TB. Symptoms were more frequently reported among contacts starting TB treatment (45/58, 78%) than among contacts initiating TPT (78/223, 35%). Most contacts with confirmed active TB were diagnosed through microbiological testing (36/58, 62%).

We summarised attrition across the early and late steps of the preventive care cascade overall and by country (figure 2). Regarding the early steps, we calculated the probability of visiting a healthcare facility and testing for TB given they currently reported TB symptoms, which was 21% (0.78×0.27) for all contacts, 32% (0.91×0.35) for contacts with HIV and 23% (0.88×0.26) for contacts <5 years. Regarding the late steps, we calculated the probability of visiting a healthcare facility, being eligible for TPT and initiating TPT, which was 4% (0.78×0.98×0.05) for all contacts, 11% (0.91×0.95×0.13) for contacts with HIV and 14% of contacts <5 years (0.88×0.99×0.16).

**Figure 2:**
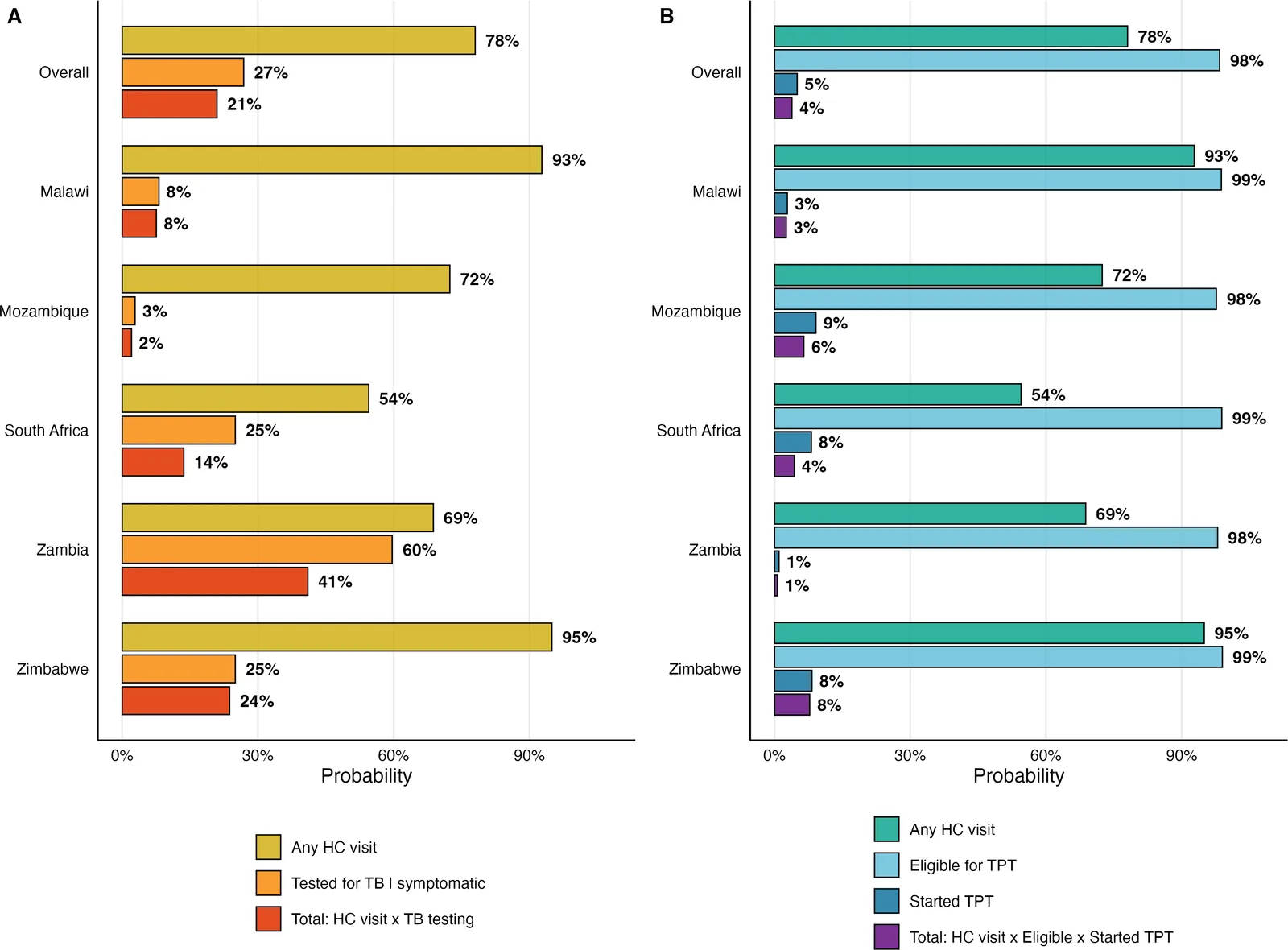
Attrition over preventive care cascade. (**A**) Proportion of contacts (probabilities, %) who visited any healthcare facility (HC) and tested for tuberculosis (TB) given they currently reported TB symptoms. The total probability for completing both steps in the early care cascade is the product of the two probabilities. (**B**) Proportion of contacts who visited any HC, were considered eligible for tuberculosis preventive treatment (TPT) and initiated TPT. The total probability for completing all three steps in the late care cascade is the product of the three probabilities.

### Determinants of care seeking

Contacts reporting current TB symptoms more often visited a healthcare facility than those not reporting symptoms (OR 8.23, 95% CI 5.09 to 13.31; online supplemental figure S1). Current TB symptoms were more often reported among contacts with HIV (OR 1.80, 95% CI 1.19 to 2.75) and prior history of TB (OR 1.70, 95% CI 1.12 to 2.58) as well as among contacts living in rural instead of urban areas (OR 2.70, 95% CI 1.69 to 4.33) (figure 3A). Children and siblings reported symptoms less often than spouses. Contacts of index patients with MDR-TB more often reported symptoms than those of index patients with drug-sensitive TB (OR 1.76, 95% CI 1.04 to 2.99).

**Figure 3:**
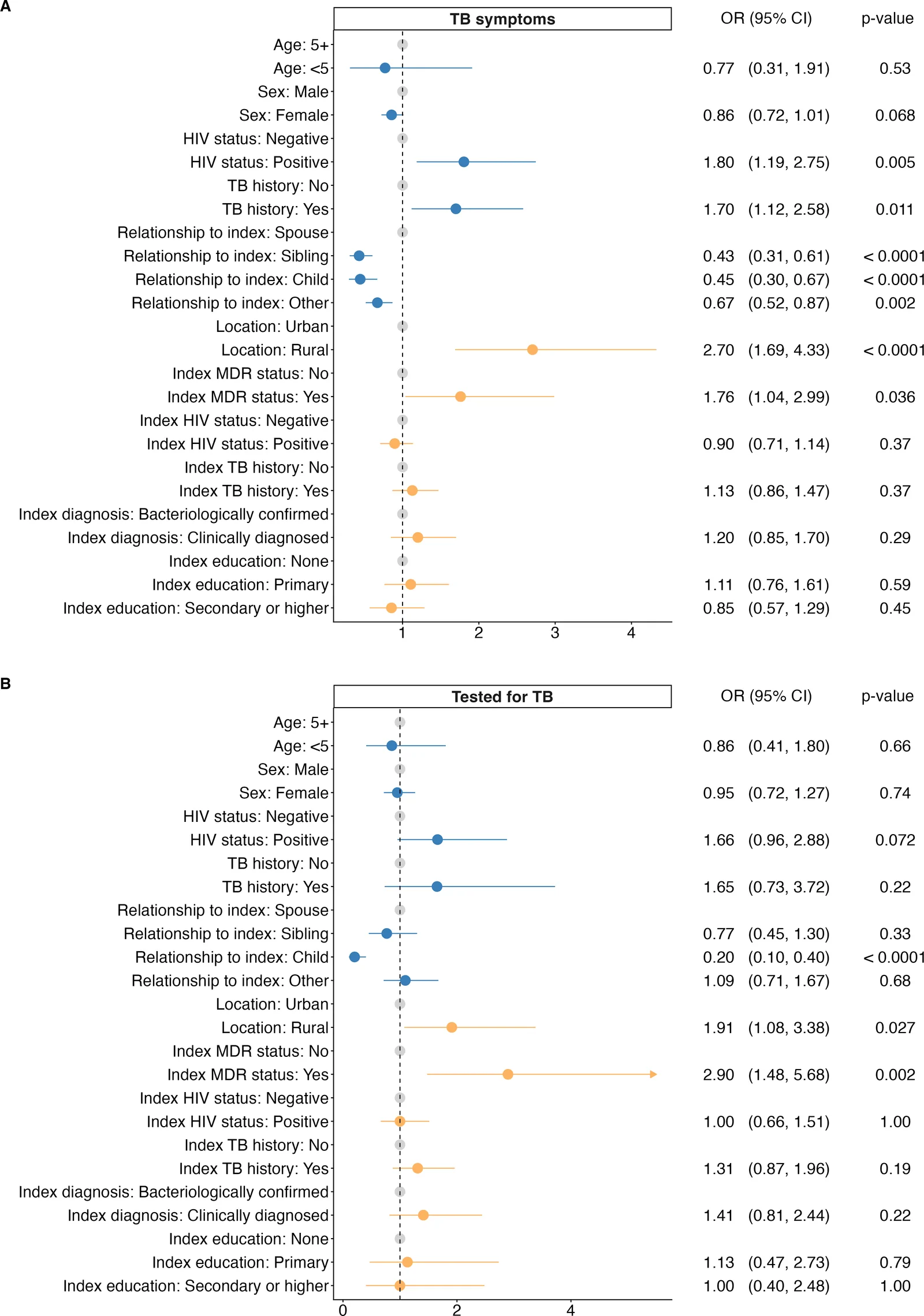
Associations of household contact and index patient characteristics with (**A**) the reporting of current TB symptoms and (**B**) microbiological TB testing among contacts visiting a healthcare facility. OR as dots and 95% CIs as lines; contact-level characteristics in blue and index-level characteristics in orange; reference categories as grey dots. MDR, multidrug-resistant; TB, tuberculosis.

Contacts with HIV tended to test more often for TB (OR 1.66, 95% CI 0.96 to 2.88), as did contacts living in rural instead of urban areas (OR 1.91, 95% CI 1.08 to 3.38) (figure 3B). Contacts of index patients with MDR-TB also tested more often for TB (OR 2.90, 95% CI 1.48 to 5.68), while children tested less often than spouses (OR 0.20, 95% CI 0.10 to 0.40). These associations were attenuated when considering only contacts reporting current TB symptoms (online supplemental figure S2).

### Determinants of TB diagnosis and TPT initiation

Contacts reporting current TB symptoms were more often diagnosed with active TB (OR 14.10, 95% CI 5.56 to 35.79), as were contacts with a history of TB (OR 14.26, 95% CI 4.73 to 42.98) (figure 4A). Contacts living in rural areas were less often diagnosed with active TB than those in urban areas (OR 0.24, 95% CI 0.12 to 0.49).

**Figure 4:**
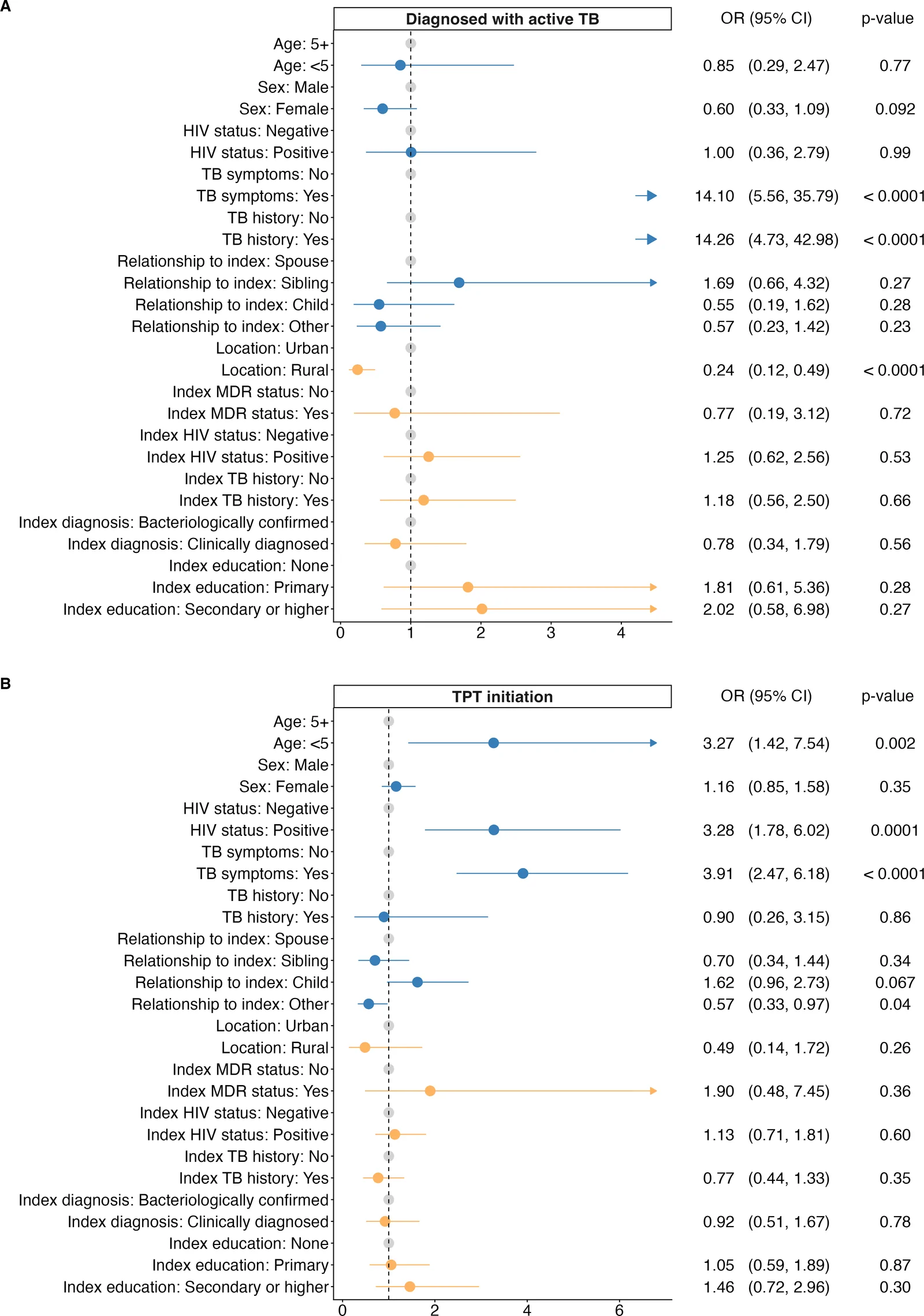
Associations of household contact and index patient characteristics with (**A**) diagnosis of active TB among contacts visiting a healthcare facility and (**B**) TPT initiation among contacts eligible for TPT. OR as dots and 95% CIs as lines; contact-level characteristics in blue and index-level characteristics in orange; reference categories as grey dots. MDR, multidrug-resistant; TB, tuberculosis; TPT, tuberculosis preventive treatment.

Among TPT-eligible contacts, TPT initiation was more frequent among people with HIV (OR 3.28, 95% CI 1.78 to 6.02) and children <5 years (OR 3.27, 95% CI 1.42 to 7.54) (figure 4B). Modelling the association with age as a continuous predictor confirmed that young children were prioritised for TPT (online supplemental figure S3). TPT initiation was also higher among contacts reporting current TB symptoms (OR 3.91, 95% CI 2.47 to 6.18), especially in the subgroups prioritised for TPT (online supplemental figure S4).

### Sensitivity analysis

Index patients recorded household contact information at multiple visits. We performed a sensitivity analysis excluding potential mismatches, which refers to newly created contacts from entries of later visits that could not be linked to entries from earlier visits, and where the reported number of household members did not increase. The preventive care cascade and the associations between outcomes and predictors were very similar when excluding potential mismatches (online supplemental figures S5 and S6).

## Discussion

We studied the preventive care cascade among household contacts of patients with TB starting treatment in five TB cohorts in the IeDEA Southern Africa region. More than 75% of household contacts visited a healthcare facility, but only 5% of eligible contacts eventually initiated TPT. Initiation was three times more likely in priority groups for TPT according to WHO guidelines, including people with HIV and children <5 years. Current TB symptoms were more frequently reported among contacts with HIV, a history of TB and with an MDR-TB index patient. Contacts reporting current TB symptoms, but who were not yet diagnosed with TB, were four times more likely to initiate TPT. Most contacts who initiated TPT already completed it, while the treatment was ongoing for all others except one contact who interrupted it and one contact with unknown status.

TPT initiation was low among household contacts of patients with TB starting treatment in our five TB cohorts in Southern Africa, far below the 90% target set by the United Nations’ High-Level Meeting in 2023 for high-risk groups such as household contacts and people with HIV.^14^ Although TPT initiation was more likely among people with HIV, the rate was still below the 40%–60% reported by a recent meta-analysis.^13^ Studies from the Southern African region also reported strong heterogeneity in TPT initiation,^15–18^ from as low as 0.5% among contacts of newly diagnosed patients with TB at a rural district hospital in the Eastern Cape, South Africa,^15^ to 25% among household contacts in Copperbelt and Lusaka, Zambia.^16^ These differences likely stem from several factors, including the study population of TPT-eligible subgroups (eg, household contacts vs people with HIV), the setting (eg, rural vs urban areas) and the characteristics of the index patient (eg, drug-sensitive vs MDR-TB patients). These factors can vary both between and within countries; for example, TPT initiation varied by several magnitudes within Malawi^17^ and Zambia.^16^

Against this wide regional variation, our overall TPT initiation places our cohorts at the lower end of the reported spectrum. How far this reflects the broader situation in the region depends on the representativeness of our sites. The IeDEA-SA clinics were not randomly sampled and may not mirror each country’s national health infrastructure, so our estimates should be generalised with caution. Several features of our study nonetheless argue against them marking an extreme low. Our cohorts span five countries and both urban and rural facilities, capturing a broad range of clinical realities rather than a single setting, and initiation was considerably higher in priority groups such as people with HIV and children <5 years. Moreover, participation in a prospective cohort, together with the structured information provided to index patients, may itself have raised awareness of TPT and increased initiation above what routine care achieves. In this case, our estimates could then have overstated rather than understated initiation.

We found that TPT initiation was higher among household contacts with HIV and children <5 years, in line with the priority groups set by the WHO.^10^ In contrast to studies from Bangladesh,^23^ Malawi,^22^ Eswatini^24^ and Kenya^25^, which found higher TPT initiation among household contacts in rural areas, we found no clear association between dwelling location and TPT initiation after adjustment for country. Because dwelling location was almost entirely determined by study site, with index patients living almost exclusively in rural areas in Mozambique and Zimbabwe and in urban areas at the other three sites, its effect could not be separated from site-level differences in TPT delivery, such as the lower workload of healthcare workers in rural areas, which may allow better quality of care.^25^

There may also be structural barriers to TPT initiation. In low-resource settings, drug or staff shortages may provide natural limitations to TPT initiation.^26 27^ Yet, even if these limitations can be resolved, both patients and healthcare workers may still be hesitant to initiate TPT. People at risk of infection with TB may be hesitant due to stigma associated with the disease, concerns about side effects and pill burden,^21 28–30^ or they have little knowledge about TPT in the first place,^26 28^ suggesting that better patient counselling as well as broader health education programmes could increase awareness about the benefits of TPT. Healthcare workers may be hesitant to prescribe TPT due to uncertainty about policy guidelines and eligibility criteria.^26 31^ People with HIV are generally eligible for TPT and could be captured through integration of TB/HIV services.^21 31 32^ Expanding TPT coverage to other groups likely necessitates systematic scale-up of contact-tracing strategies,^21 26 27^ with community-based,^24 33^ household-centred^18 34^ or digital approaches^33^ showing promising results.

We managed to reach more than 75% of household contacts and advised them to visit a healthcare facility. Related studies reported screening more than 80% of TB household contacts,^35 36^ but they achieved this through house visits^36^ or a household contact card and register for systematic screening and follow-up.^35^ Attrition along the TB preventive care cascade has been reported to be high, especially at the stage of microbiological testing.^37^ For example, the cumulative probability that a contact would be screened and evaluated was only 5% in Uganda,^38^ compared with 21% in our study. Furthermore, while a final assessment could not yet be made, most household contacts who initiated TPT had already completed it; for all others, except one patient who interrupted TPT and another with unknown status, TPT was ongoing. This contrasts with multiple studies reporting high attrition rates after TPT initiation.^39–42^ Thus, while other studies report high attrition after initiation, our findings suggest that the implementation gap is greater at TPT initiation than at completion. Nevertheless, adherence to TPT could also be improved, for example, through community-based or patient-centred approaches as well as shorter regimens.^21 43–45^ In particular, short-course regimens with isoniazid and rifapentine daily for 1 month or weekly for 3 months have similar completion rates and safety^46^ and are non-inferior to longer course regimens.^47–49^

Our study has several limitations. First, while the IeDEA sites may not be fully representative of each country’s national health infrastructure, our multicentric design across five diverse nations captures a broad spectrum of clinical realities in Southern Africa. Second, observed intercountry variations may reflect operational heterogeneity in data abstraction rather than purely epidemiological differences. We addressed this site-level variance by incorporating country-specific effects into our multivariable models. Third, longitudinal attrition was significant, likely driven by high population mobility and fluid household compositions in the region. However, such attrition is inherently part of the TB preventive care cascade, which this study aimed to quantify. Fourth, reliance on index patient reporting for household contact data may introduce inaccuracies; we mitigated this by providing index patients with structured information sheets to guide data collection. Nevertheless, some contacts may have accessed TB screening or TPT services outside the reporting system or through alternative community-based platforms not captured in our study, meaning the observed TPT uptake may be underascertained. Conversely, the study itself may have promoted TPT awareness among participating index patients and their contacts, such that TPT initiation in routine practice could be even lower than reported here. Finally, we assumed that all household contacts without active TB were eligible for TPT, but the available data did not allow us to exclude individuals with contraindications such as active liver disease, acute hepatitis or hypersensitivity to TPT drugs, which may have led us to underestimate the proportion of eligible contacts who initiated TPT.

Our study reveals a critical implementation gap in the TB preventive care cascade across Southern Africa, where despite high healthcare engagement, TPT initiation among eligible household contacts remains far below global targets to end the TB epidemic. While prioritisation of high-risk groups such as children under 5 and people with HIV is evident, the overall low uptake, compounded by low rates of microbiological testing for symptomatic contacts, underscores a missed opportunity for community-level TB control. These findings suggest that overcoming structural and sociodemographic barriers will require a shift towards more integrated, household-centred screening and the systematic scale-up of contact-tracing strategies. Ultimately, bridging these gaps is essential to reducing the regional TB burden and safeguarding the health of vulnerable household members.

## Supplementary material

10.1136/bmjgh-2026-025497 online supplemental file 1

10.1136/bmjgh-2026-025497 online supplemental file 2

## Data Availability

Data are available upon reasonable request.
