# Supplementary material for: Tuberculosis preventive treatment and care cascade among household contacts of patients starting tuberculosis treatment in Southern Africa: a multicountry cohort analysis
Source: BMJ Glob Health. 2026 Oct 30;11(10):e025497. doi: 10.1136/bmjgh-2026-025497 (PMC13723798; doi:10.1136/bmjgh-2026-025497)
Supplement: Supplementary file 1 [file bmjgh-11-10-s001.docx]

### BMJ Global Health Author Reflexivity Statement

Adapted from Morton, B., Vercueil, A., Masekela, R., Heinz, E., Reimer, L., Saleh, S., Kalinga, C., Seekles, M., Biccard, B., Chakaya, J., Abimbola, S., Obasi, A. and Oriyo, N. (2022), Consensus statement on measures to promote equitable authorship in the publication of research from international partnerships. Anaesthesia, 77: 264-276. <https://doi.org/10.1111/anae.15597>

| **Study conceptualisation** | |
| --- | --- |
| 1. How does this study address local research and policy priorities? | We discussed the objectives, the content, and the study design with the relevant working groups within the global International epidemiology Databases to Evaluate AIDS (IeDEA) consortium, including the Tuberculosis and Lung Health working groups. The study addresses a recognised regional priority by quantifying gaps in the tuberculosis preventive care cascade among household contacts of TB patients across five high-burden Southern African countries (Malawi, Mozambique, South Africa, Zambia and Zimbabwe). |
| 2. How were local researchers involved in study design? | Through the relevant working groups within the global IeDEA consortium, in particular the IeDEA Southern Africa (IeDEA-SA) region, and through the investigators at the five participating TB/HIV care clinics, who contributed to the study design and to data collection. |
| **Research management** | |
| 3. How has funding been used to support the local research team(s)? | The global IeDEA consortium supports the regional data centres and the local sites participating in the cohort. The present study was supported by the US National Institute of Allergy and Infectious Diseases (NIAID/NIH; award U01AI069924), which funds the IeDEA-SA regional data centre and participating sites, and by the Swiss National Science Foundation (grant P500PM_230473). |
| **Data acquisition and analysis** | |
| 4. How are research staff who conducted data collection acknowledged? | In line with international standards, research staff are named as co-authors according to IeDEA’s Standard Operating Procedure (SOP), or in the Acknowledgements section. Investigators, clinicians and data managers at the participating sites who carried out contact tracing and data collection are named as co-authors or listed under their respective IeDEA-SA site in the Acknowledgements section. |
| 5. How have members of the research partnership been provided with access to study data? | Data are available upon reasonable request and, after evaluation, are provided based on a standardised process within the IeDEA-SA network, which requires the submission and approval of a concept note by the regional Steering Groups. |
| 6. How were data used to develop analytical skills within the partnership? | A junior biostatistician conducted the analysis under the supervision and training of a senior biostatistician, developing analytical skills relevant to this study and transferable to other projects within the IeDEA consortium. Both also contributed to building analytical skills among other statisticians in the consortium, especially in lower-income countries in Southern Africa, through the provision of statistical support. |
| **Data interpretation** | |
| 7. How have research partners collaborated in interpreting study data? | The preliminary results were discussed in the relevant working groups of the IeDEA consortium. Draft versions of the manuscript were circulated among the co-authors, including the investigators at the participating sites, and the final version was approved in accordance with the IeDEA-SA review process. |
| **Drafting and revising for intellectual content** | |
| 8. How were research partners supported to develop writing skills? | There were several rounds of reviews by the co-authors, during which comments could be made and writing skills developed. The IeDEA consortium also provides grant-writing courses and specialised epidemiology training (Fogarty-IeDEA Mentorship Programme, FIMP). |
| 9. How will research products be shared to address local needs? | We will disseminate the results of this study to the participating TB/HIV care clinics and our broader research networks. |
| **Authorship** | |
| 10. How is the leadership, contribution and ownership of this work by LMIC researchers recognised within the authorship? | Authorship slots are managed according to IeDEA’s SOP, which aims to include as many LMIC researchers as possible on the author list. All other contributors are listed in an extended Acknowledgements section. |
| 11. How have early career researchers across the partnership been included within the authorship team? | Several early career researchers are listed as co-authors. The IeDEA regions are responsible for managing this process, as they are familiar with the research environment and aware of the early career researchers who have contributed. |
| 12. How has gender balance been addressed within the authorship? | Nine out of thirteen authors are women, including the lead author of the study. |
| **Training** | |
| 13. How has the project contributed to training of LMIC researchers? | There were several rounds of reviews by the co-authors, which enabled them to develop their writing skills and gain experience by providing feedback. The IeDEA consortium also provides grant-writing courses and specialised epidemiology training (Fogarty-IeDEA Mentorship Programme, FIMP). |
| **Infrastructure** | |
| 14. How has the project contributed to improvements in local infrastructure? | The global IeDEA consortium supports regional data centres and local sites that participate in the cohort and in ongoing data collection. |
| **Governance** | |
| 15. What safeguarding procedures were used to protect local study participants and researchers? | This was a prospective cohort study involving human participants. All participating sites obtained approval from their local institutional review boards or ethics committees, and the project was additionally approved by the Cantonal Ethics Committee of Bern, Switzerland (reference no. PB_2016-00273). All study participants gave written informed consent. |
