# Supplementary material for: Tuberculosis preventive treatment and care cascade among household contacts of patients starting tuberculosis treatment in Southern Africa: a multicountry cohort analysis
Source: BMJ Glob Health. 2026 Oct 30;11(10):e025497. doi: 10.1136/bmjgh-2026-025497 (PMC13723798; doi:10.1136/bmjgh-2026-025497)
Supplement: Supplementary file 2 [file bmjgh-11-10-s002.docx]

**Supplementary Material**

**TEXT A: Linking household contact information across time points**

Household contact information was recorded at four time points via index TB patients as they came for a visit at the start of treatment, end of treatment, and six- and twelve-months post TB treatment. Since a unique personal identifier was missing to link contact information across time points, we used an algorithmic procedure to identify entries of the same individual.

Contacts were matched within the same household across time points using a scoring system based on stable person attributes. The scores were as follows:

| - Exact match on date of birth: | 3 points |
| --- | --- |
| - Relationship recorded as spouse: | 3 points |
| - Match on year and month of birth only: | 2 points |
| - Same relationship to the index patient: | 2 points |
| - Exact match on initials: | 2 points |
| - Same initials with reversed letter order: | 1 point |
| - Same sex: | 1 point |

The matching was performed separately within each household and sequentially across time points. At baseline, we assigned a unique, household-specific contact ID. We compared entries from the end of treatment to baseline entries and calculated the similarity scores, ranking candidates from the highest to the lowest score. An end-of-treatment entry was linked to a baseline entry if the score was ≥4 points. If multiple candidates exceeded this threshold, the candidate with the highest score was selected. If there was a tie, the first recorded candidate was chosen. A new contact ID was assigned to entries that could not be linked with any baseline entry.

The matching procedure was repeated for the entries from post treatment visits. The contact ID list was updated after each round: (i) contacts from previous visits that were linked, (ii) contacts from previous visits that were not linked, and (iii) newly created contact entries that could not be linked.

Contact information of linked entries was summarized across time points as follows. First, missing information was imputed. For example, if HIV status was missing in the baseline entry of a contact but recorded in the linked end-of-treatment entry, we assigned the HIV status recorded at the end-of-treatment visit for this contact. Second, we prioritized “Yes” over “No” responses. For example, if a contact’s baseline entry indicated no healthcare visit but the end-of-treatment visit did, we assigned a healthcare visit for this contact. Note that stable contact information (age, sex and relationship to the index case) was used to link entries, thus any inconsistencies in these attributes after matching were extremely rare and resolved through manual inspection.

We examined the results from the matching procedure. Overall, there were 5,799 follow-up records, of which 3,938 (68%) entries could be linked to an existing contact ID. The remaining 1,861 (32%) entries could not be linked and were therefore assigned a new contact ID. Of these 1,861 newly created contacts, 1,164 (63%) could not be explained by an increase in the number of household members. These contacts were potential mismatches due to either insufficient or inconsistent identifying information, or substantial differences in recorded characteristics across timepoints. In the main text, we performed a sensitivity analysis where we excluded these potential mismatches. We also obtained 172/5,799 (3%) entries with ties and a score ≥4 points. Due to their small number, we refrained from an additional sensitivity analysis to assess their influence.

**Supplementary Table S1: Summary of contact tracing activities.**

|  | **Overall**  N = 1,369^1^ | **Malawi**  N = 258^1^ | **Mozambique**  N = 300^1^ | **South Africa**  N = 196^1^ | **Zambia**  N = 360^1^ | **Zimbabwe**  N = 255^1^ |
| --- | --- | --- | --- | --- | --- | --- |
| **Index contacted through formal tracing** | | | | | | |
| No | 837 (62%) | 256 (99%) | 2 (0.7%) | 162 (92%) | 248 (70%) | 169 (66%) |
| Yes | 509 (38%) | 2 (0.8%) | 298 (99%) | 15 (8.5%) | 108 (30%) | 86 (34%) |
| Missing | 23 | 0 | 0 | 19 | 4 | 0 |
| **Who contacted the index** | | | | | | |
| Clinic staff | 295 (35%) | 9 (4.2%) | 21 (7.0%) | 42 (93%) | 136 (71%) | 87 (99%) |
| External organization | 280 (33%) | 0 (0%) | 279 (93%) | 0 (0%) | 1 (0.5%) | 0 (0%) |
| Others | 262 (31%) | 203 (96%) | 0 (0%) | 3 (6.7%) | 55 (29%) | 1 (1.1%) |
| Missing | 532 | 46 | 0 | 151 | 168 | 167 |
| **Any contact tracing after diagnosis** | | | | | | |
| No | 479 (37%) | 72 (28%) | 1 (0.3%) | 127 (89%) | 113 (32%) | 166 (66%) |
| Yes | 831 (63%) | 186 (72%) | 299 (100%) | 16 (11%) | 244 (68%) | 86 (34%) |
| Missing | 59 | 0 | 0 | 53 | 3 | 3 |
| **Contacts advised visiting clinic** | | | | | | |
| No | 280 (22%) | 64 (25%) | 3 (1.0%) | 116 (79%) | 43 (12%) | 54 (23%) |
| Yes | 1,019 (78%) | 194 (75%) | 297 (99%) | 30 (21%) | 313 (88%) | 185 (77%) |
| Missing | 70 | 0 | 0 | 50 | 4 | 16 |
| ^1^ Contact tracing activities for index patients with at least one recorded household contact; among 1,438 index patients, 1,369 (95%) had at least one recorded household contact. | | | | | | |

**Supplementary Figure S1: Associations of household contact and index patient characteristics with visits of contacts to a healthcare facility.** Odds ratios (OR) as dots and 95%-confidence intervals as lines; contact-level characteristics in blue and index-level characteristics in orange; reference categories as grey dots.

**Supplementary Figure S2: Associations of household contact and index patient characteristics with microbiological TB testing among contacts reporting current TB symptoms.** Odds ratios (OR) as dots and 95%-confidence intervals as lines; contact-level characteristics in blue and index-level characteristics in orange; reference categories as grey dots.

**Supplementary Figure S3: Association between age and TPT initiation.** Shown is the probability of TPT initiation across the entire age range observed among household contacts. A possible non-linear association for age was assessed by using age as a continuous predictor and modelling its effects with a cubic spline with 3 degrees of freedom. The same multivariable logistic regression model as in the main analysis was used: country fixed effects, standard errors clustered at the household level and contact and index-level covariates. The probability of TPT initiation was computed by averaging the model-predicted probabilities across contacts. Marginal probability as line and 95% confidence interval as shaded area.

**Supplementary Figure S4: Associations of household contact and index patient characteristics with TPT initiation among priority groups (contacts with HIV and children <5 years).** Odds ratios (OR) as dots and 95%-confidence intervals as lines; contact-level characteristics in blue and index-level characteristics in orange; reference categories as grey dots.

**Supplementary Figure S5:** **Sensitivity analysis showing the TB preventive care cascade among household contacts of index patients when excluding potentially mismatched contacts.** **(A)** Proportion of all household contacts per step in the TPT cascade. **(B)** Flows among only household contacts initiating TPT or TB treatment. Steps include visiting any healthcare facility (HC), reporting current TB symptoms, testing for TB (microbiological testing), receiving a TB diagnosis, and initiating TPT or TB treatment. TPT eligibility: all contacts visiting a healthcare facility were considered eligible for TPT, except contacts with confirmed, latent or suspected TB cases. Mismatched contacts refer to newly created contacts from entries recorded at later visits of the index patient that could not be linked to earlier entries and where the newly created contact did not align with an increase in the recorded number of household members.

**Supplementary Figure S6: Sensitivity analysis showing the associations of household contact and index patient characteristics with care cascade outcomes after excluding potentially mismatched contacts. (A)** TB symptom reporting, **(B)** microbiological TB testing, **(C)** diagnosis with active TB, and **(D)** TPT initiation. Odds ratios (OR) as dots and 95%-confidence intervals as lines; contact-level characteristics in blue and index-level characteristics in orange; reference categories as grey dots. Mismatched contacts refer to newly created contacts from entries recorded at later visits of the index patient that could not be linked to earlier entries and where the newly created contact did not align with an increase in the recorded number of household members.
